# Symptomatic Periarticular Fluid Collection After Total Hip Arthroplasty: Septic or Aseptic Complication? A Case Report and Literature Review

**DOI:** 10.3390/reports8040214

**Published:** 2025-10-24

**Authors:** Dan Vlad Stanescu, Jenel Marian Patrascu, Ahmed Abu-Awwad, Alina Simona Abu-Awwad, Jenel Marian Patrascu

**Affiliations:** 1 Department XV-Discipline of Orthopedics-Traumatology, “Victor Babes” University of Medicine and Pharmacy, Eftimie Murgu Square, No. 2, 300041 Timisoara, Romania; dan.stanescu@umft.ro (D.V.S.); jenel.patrascu@umft.ro (J.M.P.J.); patrascu.jenel@umft.ro (J.M.P.); 2 2nd Clinic of Orthopedics and Traumatology, County Emergency Hospital ‘Pius Branzeu’, Nr. 2, 300041 Timisoara, Romania; 3 ‘Professor Teodor Sora’ Research Center, U.M.F., Nr. 2, 300041 Timisoara, Romania; 4 1st Clinic of Obstetrics and Gynecology, “Pius Brinzeu” County Clinical Emergency Hospital, 300723 Timisoara, Romania; alina.abuawwad@umft.ro; 5 Department of Obstetrics and Gynecology, Faculty of Medicine, “Victor Babes” University of Medicine and Pharmacy, 300041 Timisoara, Romania

**Keywords:** total hip arthroplasty, adverse reaction to metal debris, periprosthetic joint infection, metal-on-polyethylene, ARMD, ALVAL, metallosis, revision total hip arthroplasty

## Abstract

**Background and Clinical Significance**: Adverse reactions to metal debris (ARMD) are a rare but increasingly recognized complication following total hip arthroplasty (THA), with some studies suggesting upwards of 5% of metal-on-metal (MoM) and 3% of metal-on-polyethylene (MoP) prostheses being attributed to this. Historically, metallosis due to MoM implant design was the primary cause of ARMD. However, ARMD can also arise in metal-on-polyethylene (MoP) prostheses due to trunnionosis, which involves wear and corrosion at the modular femoral head–neck interface. Clinically, ARMD can resemble periprosthetic joint infection (PJI), complicating both diagnosis and management. **Case Presentation**: We present the case of a 40-year-old female with a history of systemic degenerative joint disease with bilateral MoP THAs who developed progressive pain and swelling in the upper left thigh, in which the prosthesis was first put in 22 years prior. The patient presented initially in a vascular surgery department for an infected iliopsoas cyst communicating with the hip where she had received surgery 2 years prior. The symptomatology reoccurred, and imaging revealed a large mass near the prosthesis and elevated inflammatory markers. Intraoperatively, a large volume of sero-purulent fluid was encountered, prompting a diagnostic workup for PJI. All cultures returned negative, and histopathology revealed macrophage-dominant infiltration with metallic debris, consistent with ARMD. After infection was definitively excluded, a revision THA was performed with an exchange of all modular components. The patient recovered without complications, and at six months follow-up, she demonstrated stable implant positioning, restored function, and no recurrence of symptoms. **Conclusions**: This case highlights the diagnostic complexity of PJI in joint arthroplasty and reveals the importance of a protocol-driven approach to exclude it prior to surgical revision. As the incidence of trunnion-related failure becomes more recognized in the literature, clinicians must consider ARMD in the differential diagnosis of late THA complications. Appropriate diagnosis is essential for guiding treatment and avoiding unnecessary complications, morbidity, and treatment related side-effects.

## 1. Introduction and Clinical Significance

Adverse reactions to metal debris (ARMD) represent a rare complication associated with total hip arthroplasty. Historically, the prevalence of hip metallosis, with some studies reporting it to be as high as high as 5% [1] in all MoM prostheses, was higher when metal-on-metal prostheses were more widely used [2]. In contemporary practice, the predominance of metal-on-polyethylene prostheses has substantially reduced the release of metal ions, thereby decreasing clinically significant cases of metallosis. Another contributor to metal ion release is trunnionosis, which refers to wear at the femoral head–neck interface. It is estimated that up to 2% of all THA patients can be affected by trunnionosis and reports have demonstrated an incidence ranging from 0.7 to 3% for all revision THAs being performed for this reason [3].

There are several reported risk factors for the development of ARMD, including femoral head size, male sex, and inflammatory disease; however, the etiology remains poorly understood [4].

The most important differential diagnosis for (ARMD) is periprosthetic joint infection (PJI) [5]. PJI can manifest a wide spectrum of clinical presentations, ranging from acute fulminant infection with systemic symptoms to vague symptoms that can show years after the index procedure. Despite advances in aseptic techniques, PJI remains one of the leading causes of rTHA, with studies showing 13% of all revisions being performed for this reason [6]. The clinical presentation of these conditions can be similar, and radiographic findings are often inconclusive. Therefore, advanced diagnostic testing is essential for excluding infection and confirming the diagnosis of ARMD [7]. Investigations such as serum metal ion measurements and MARS-MRIs have demonstrated utility in detecting early and asymptomatic ARMD [8].

In the present case, the patient presented with a mass in the left upper leg, a history of bilateral total hip arthroplasties (THAs), and systemic inflammatory disease, alongside a prior episode of an infected periarterial cyst communicating with the prosthesis. A revision THA was subsequently planned. Periarticular fluid collection may arise due to a variety of etiologies, with one study reporting 17% of native joint aspirations revealing septic arthritis as the underlying cause [9], while other etiologies can include inflammatory arthropathies (e.g., rheumatoid arthritis, ankylosing spondylitis, and reactive arthritis), non-inflammatory conditions (e.g., amyloidosis, osteoarthritis, and systemic sclerosis), and hemorrhagic causes (e.g., coagulopathy, anticoagulant therapy, thrombocytopenia, or trauma) [10].

The research question guiding this report was formulated in PICO format as follows: In patients with painful total hip arthroplasty and suspected ARMD (P), do advanced diagnostic tools such as serum metal ion levels, MRI, and histological examination (I) compared with conventional investigations like plain radiographs and standard laboratory markers (C) improve the accuracy of a diagnosis, distinguishing it from other causes (infection or loosening) and thus improving the long-term outcome following rTHA?

## 2. Materials and Methods

This study combined a case report with a narrative review of the existing literature on adverse reactions to metal debris, trunnionosis, and metallosis following THA.

For the case report, data were retrospectively collected from the patient’s medical records, imaging studies, surgical notes, and histopathological reports. The patient provided written informed consent for the use of her clinical information and images. The clinical presentation, laboratory findings, intraoperative observations, microbiological cultures, histological results, and postoperative outcomes were documented. All procedures were performed in accordance with the institutional ethical standards and the Declaration of Helsinki.

For the literature review, a comprehensive search was performed using PubMed, Embase, and Scopus, covering articles published up to March 2025. The search terms included “trunnionosis”, “metallosis”, “adverse reaction to metal debris”, “pseudotumor”, “ALVAL”, and “aseptic lymphocyte-dominant vasculitis-associated lesion”. Relevant articles were identified and screened by title. Of these, articles were selected based on their relevance to the clinical presentation, diagnosis, treatment, and outcomes of the ARMD, trunnionosis, and metallosis in the context of THA. Both original research studies (prospective and retrospective studies, case series, and case reports) and review articles published in English were considered. The studies were chosen to provide clinically meaningful insights rather than an exhaustive quantitative synthesis. Studies lacking sufficient clinical detail were excluded.

Data extracted from the literature included study design, patient demographics, implant characteristics, clinical presentation, diagnostic strategies, treatment approaches, and outcomes. Owing to the heterogeneity of study designs and reported outcomes, a systematic review was not performed. Instead, the findings were synthesized narratively to provide a clinical context for the present case.

## 3. Results

### 3.1. Background

Trunnionosis is characterized by wear at the femoral head–neck interface and has recently been recognized as an increasingly important contributor to revision total hip arthroplasty (rTHA) [3]. Some studies have suggested that up to 3% of all revision THAs are attributable to trunnionosis. Adverse reactions to metal debris (ARMD) in metal-on-polyethylene (MoP) THAs may present in various forms, ranging from superficial manifestations such as cutaneous rashes to pain mimicking trochanteric bursitis, as well as localized swelling.

Metallosis, defined as aseptic fibrosis, local necrosis, or loosening of a device due to the release of metal-wear debris into adjacent tissues, results from of metallic erosion and the subsequent debris release, which triggers a significant cytokine release from inflammatory cells. The diagnosis of metallosis is relatively rare, with an estimated prevalence of up to 5% [11]. ARMD is not exclusive to metal-on-metal (MoM) implants, and some studies have reported that up to of 7.5% of all ARMD cases occur in non-MoM prostheses [12]. One registry-based study reported the incidence of revision solely attributed to ARMD in non-MoM prosthesis being 0.1% [13]. The percentage of revisions, which was between 1% and 3%, was lower than what had previously been reported in systematic reviews. This may not reflect broad community use but rather implant-specific problems, the use of certain implants at a hospital level, or different definitions of ARMD. Another registry-based study reported an estimated revision rate due to ARMD as low as 0.7% [14].

The incidence of pseudotumors and acute lymphocytic vasculitis-associated lesions (ALVALs) in MoM THA as been reported to range from 0 to 6.5%, with a pooled estimate of 0.6% [15], with one study showing that 9% of hips presented with ALVAL on MRI examinations [2].

Histologically, ARMD is a spectrum of changes from pure metallosis, ARMD, and granulomatous inflammation. ALVAL, a distinctive inflammatory response seen in ARMD, is a precursor of lymphoid neogenesis which has been documented in other chronic inflammatory conditions and is most likely the reason hip arthroplasties fail [16].

### 3.2. History

The etiology and pathogenesis of trunnionosis remain poorly understood, with various risk factors and potential mechanisms proposed as contributing causes. These causes can be broadly divided into mechanical factors, patient-related factors, and surgeon-related factors.

Mechanical factors include micromotion and corrosion. Micromotion is an inherent consequence of the modular design of contemporary implants. Most metal alloys used in orthopedic surgery are susceptible to the formation of a passive film of metal oxides that inhibits further oxidation. This micromotion causes the oxide layers to fracture, exposing the underlying metal. The cyclical processes of oxidation and reduction facilitate the development of oxidation currents, generate wear particles, and promote the onset of corrosion [17].

Patient-related factors include body mass index (BMI), activity level, anatomical factors, and medical history. A high BMI is a known risk factor for early loosening, wear, and revision of any THA prosthesis [4]. Activity levels vary significantly among individuals; however, it is generally observed that younger patients exhibit higher activity levels, thereby increasing the total amount of stress cycles prostheses go through.

Surgeon-related factors include the quality of fixation at the head–neck junction, with soft tissue interposition and the force applied being critical elements associated with loosening [18].

The management of symptomatic ARMD is entirely surgical, involving debridement, excision of affected tissue, revision of any loose or damaged components, and the use of a ceramic head.

### 3.3. Case Presentation

The patient presented to the clinic with swelling and pain in the upper left leg, exhibiting progressive symptoms over a duration of two years.

#### 3.3.1. Patient Demographics

The patient was a 40-year-old female with a history of comorbidities, including systemic arthritis, scleroderma, and chronic venous insufficiency. The patient was under treatment with antihipertensive medication, antiplatelet drugs, and antiinflamatory drugs. The patient was then under no DMARDs (disease-modifying antirheumatic drugs) or corticotherapy.

#### 3.3.2. Patient History

The patient had a documented history of HLA-negative idiopathic juvenile arthritis and scleroderma. She underwent left total hip arthroplasty in 2002 and right total hip arthroplasty in 2018 at a different medical center, both with metal-on-polyethylene prostheses.

**Figure 1:**
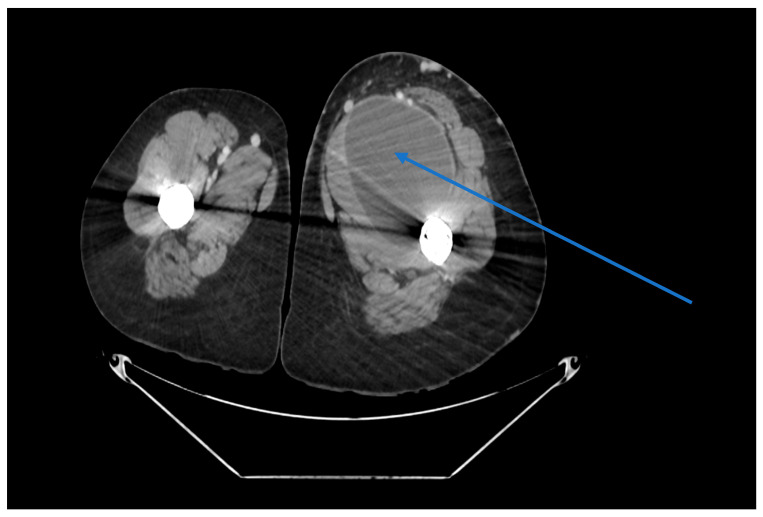
Angio-CT, axial plane, hip region. Marked by a blue arrow, large cystic lesion with fluid content is shown communicating with the hip-joint capsule.

**Figure 2:**
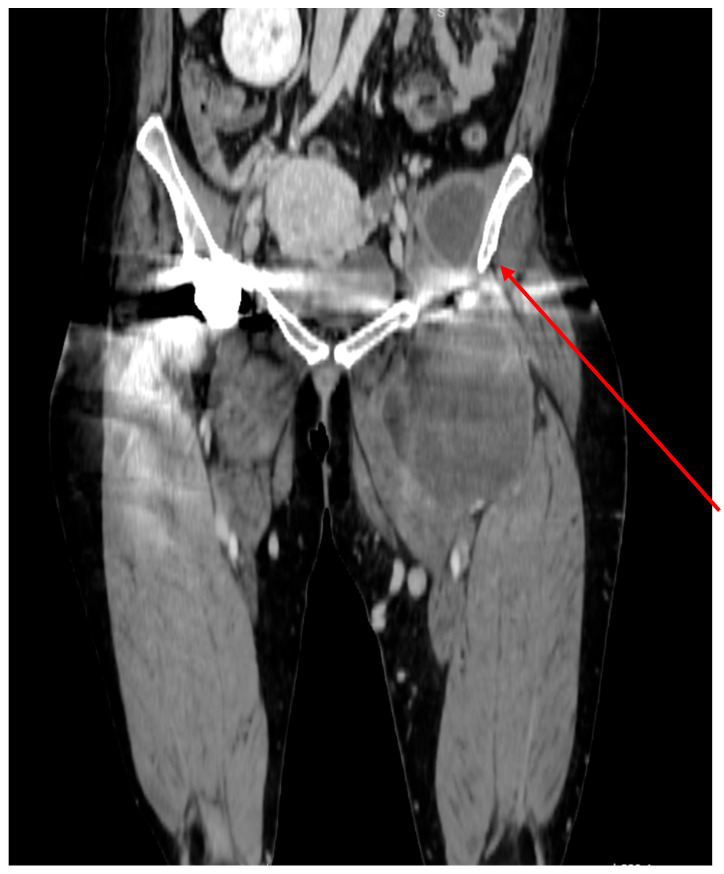
Angio-CT, coronal plane, hip region. Marked by a red arrow, fluid collection was found in the left hemi-pelvis, adjacent to the medial side of the ilium.

In 2023, the patient was admitted to the vascular surgery ward with a complicated infected cyst involving the retroperitoneal space, iliopsoas region, and inner thigh, measuring 22 × 9.2 × 3.4 cm and bordering the femoral artery. Figure 1 and Figure 2 show the original CT images. At the time of admission, the CT demonstrated communication between the cyst and the hip capsule, but in the absence of evidence for a primary infection site, the decision was made to retain the prosthesis in situ. The abscess was successfully drained, the iliac artery was sutured, and the patient was discharged without any symptoms or significant complications.

#### 3.3.3. Subjective and Objective Complaints

The patient presented to the orthopedic clinic with complaints of pain accompanied by visible swelling and a tumor-like mass in the upper left hip region. Upon clinical examination, there was no significant reduction in range of motion and no evidence of hip impingement.

#### 3.3.4. Paraclinical and Imaging Findings

**Figure 3:**
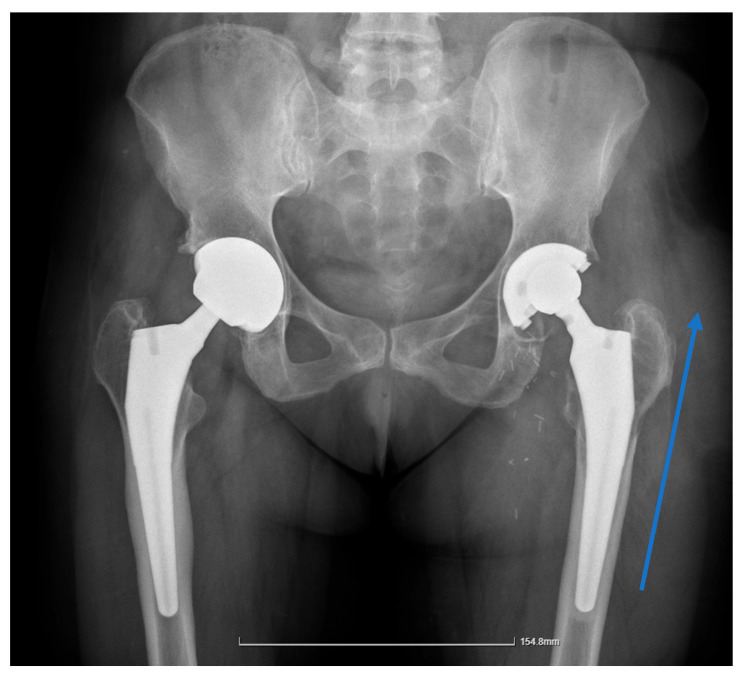
X-Ray of the pelvic area showing bilateral uncemented THA, an area of osteolysis on the acetabular section of the left leg, and the eccentric positioning of the femoral head within the cup due to polyethylene wear, with no signs of loosening in the stem. The arrow indicates a relatively well-defined area of increased radio-opacity arising from the left greater trochanteric region, suggestive of a soft-tissue mass and later confirmed operatively as fluid collection.

The X-ray revealed an area of lucency in the acetabular cup region of the implant. The initial assessment suggested the femoral stem was well fixed. Laboratory investigations demonstrated an increase in inflammatory markers (ESR = 40 mm/1 h and CRP = 30 mg/L) without associated lymphocytosis. Figure 3 shows the pelvic X-ray obtained on admission.

The original implant was a Zimmer Taperloc (Warsaw, IN, USA)^®^ System consisting of uncemented femoral and acetabular components, with a metal-on-polyethylene articulation and a 28 mm femoral head.

### 3.4. Differential Diagnosis

The initial differential diagnosis included aseptic loosening of the acetabular component, a periprosthetic joint infection, recurrence of the cyst, a localized reaction to the implant, suture material or materials used in the prior surgical intervention, and lymphatic drainage insufficiency. The patient’s history was blended by a significant vascular surgery that further jeopardized the integrity of the local vascular network. Serum metal ion levels, despite being of great use in establishing a definite diagnosis, were not routinely performed in our institution and had not been evaluated pre-operation.

Culture-negative PJI is especially difficult to treat as microbe-specific antibiotics are difficult to initiate. The prevalence of suspected culture-negative PJI is between 7% and 42%, with most studies reporting a rate of between 10% and 20% [5,19,20,21].

**Figure 4:**
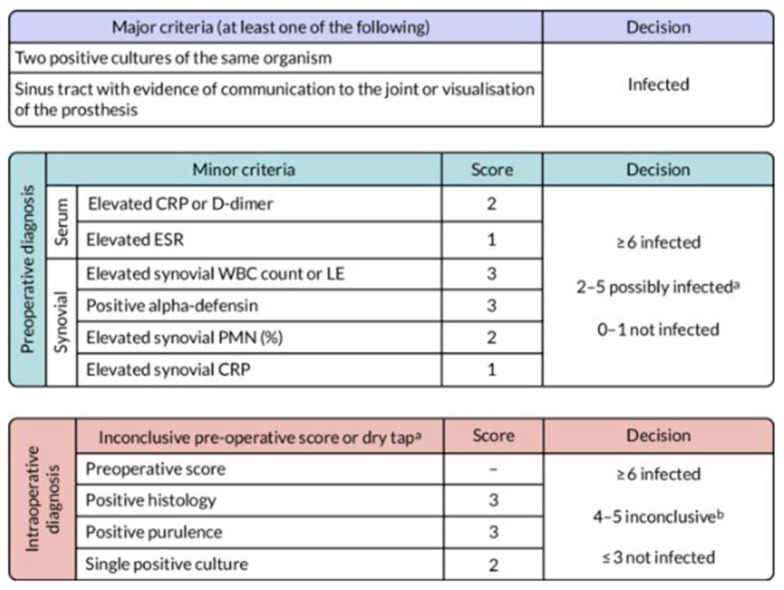
MSIS major criteria for PJI. a: For patients with inconclusive minor criteria, operative criteria can also be used to fulfill definition for PJI; b: consider further molecular diagnostics such as next-generation sequencing" per the article original source.

The Musculoskeletal Infection Society’s (MSIS’s) definition of PJI consists of two major criteria, and six minor with four or more would indicate PJI [22] Figure 4.

By this definition of PJI, the patient had a preoperative score of 3 (“possibly infected”), while the intraoperative diagnosis showed a score of 3 (“not infected”). If any of the intraoperative tests (alpha-defensin, cultures, and histology) had returned positive results, the total MSIS score would have reached 6, thereby establishing a definitive diagnosis of PJI.

### 3.5. Treatment

The patient was prepared for surgery with the intention of removing the implant and replacing the entire prosthesis. The patient was intubated and placed under general anesthesia, as opposed to epidural anesthesia, due to the presence of severe spine degenerative disease.

An incision was made parallel to the previous surgical scar, followed by soft tissue dissection and exposure of the fascia lata. Upon incising the fascia lata, a large volume of serous-purulent fluid, estimated to be between 500 mL and 750 mL, was drained. This substantial accumulation of fluid was classified as a rare form of fluid pseudotumor, and the purulent characteristics prompted the surgical team to alter the surgical plan [23].

Annex 1 presents an intraoperative Video S1 documenting the drainage of the fluid collection.

At this point, the operative strategy was revised. Multiple microbiological cultures were obtained from both the liquid and the prosthetic chamber, and tissue samples were collected for histopathological analysis. The stability of the prosthesis was evaluated, and the incision was then closed. Given the high stability of the prosthesis, the decision was made to retain the implant in situ until the results of microbiological cultures and histopathological analysis were available in order to establish a definitive diagnosis. The protocols for PJI were initiated. The cultures returned were sterile. Subsequent differential diagnoses were made, including culture-negative PJI, a local tissue reaction, and a lymphocele as possible diagnoses.

**Figure 5:**
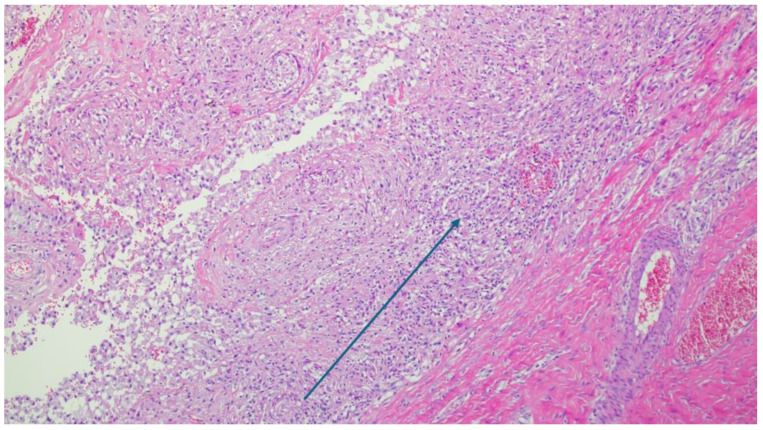
Microscopy image with H and E coloration depicting chronic inflammatory histiomacrophagic cells. The arrow is pointing at large accumulation of mononuclear cells (×10 magnification).

**Figure 6:**
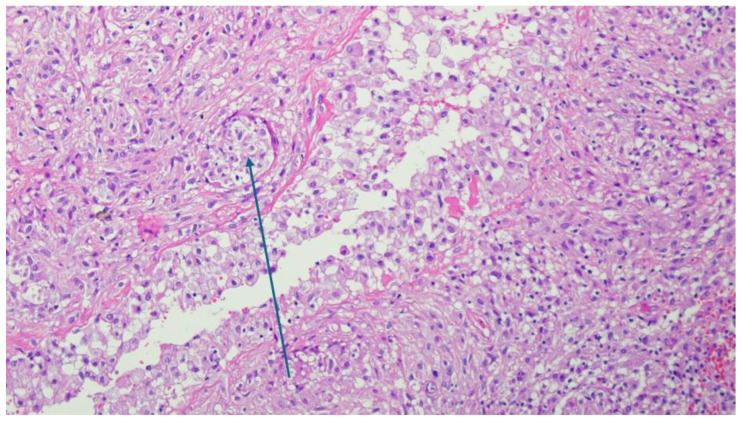
Microscopy image with H and E coloration showing the diffuse inflammatory areas. Arrow pointing at small macrophagic-rich granulomas (×20 magnification).

**Figure 7:**
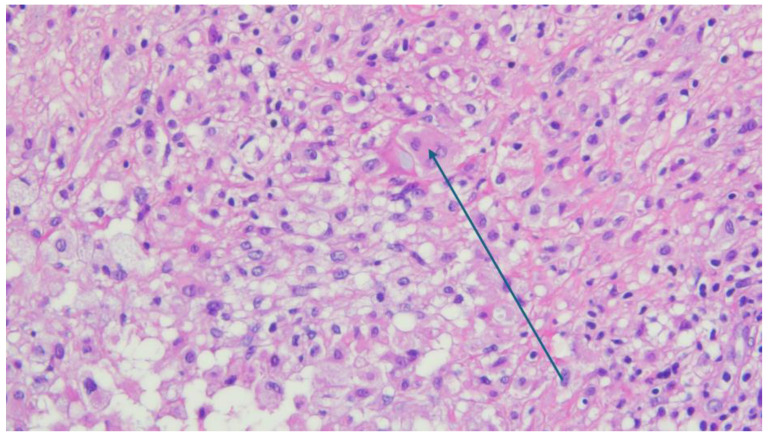
Microscopy image with H and E coloration showing the chronic mononuclear inflammatory reaction with a xantomathous pattern. The arrow indicates a giant, multinucleated resorption cell associated with weakly basophilic exogenous material (×40 magnification).

A histopathological examination revealed a non-specific granulomatous inflammatory process with diffuse areas in the form of small, better-defined macrophagic granulomas with small, slit-like spaces bordered by xanthomatous macrophages with rare PMN infiltrates, as shown in Figure 5, Figure 6 and Figure 7. Microscopy identified basophilic exogenous debris with predominant macrophagic infiltrates, consistent with a chronic inflammatory response to foreign material.

Based on a pathology report indicating an immune-mediated reaction to foreign material, negative microbiological cultures, and satisfactory wound healing, a definitive diagnosis of ARMD was established. A revision hip arthroplasty with a complete component exchange was selected as the treatment of choice.

**Figure 8:**
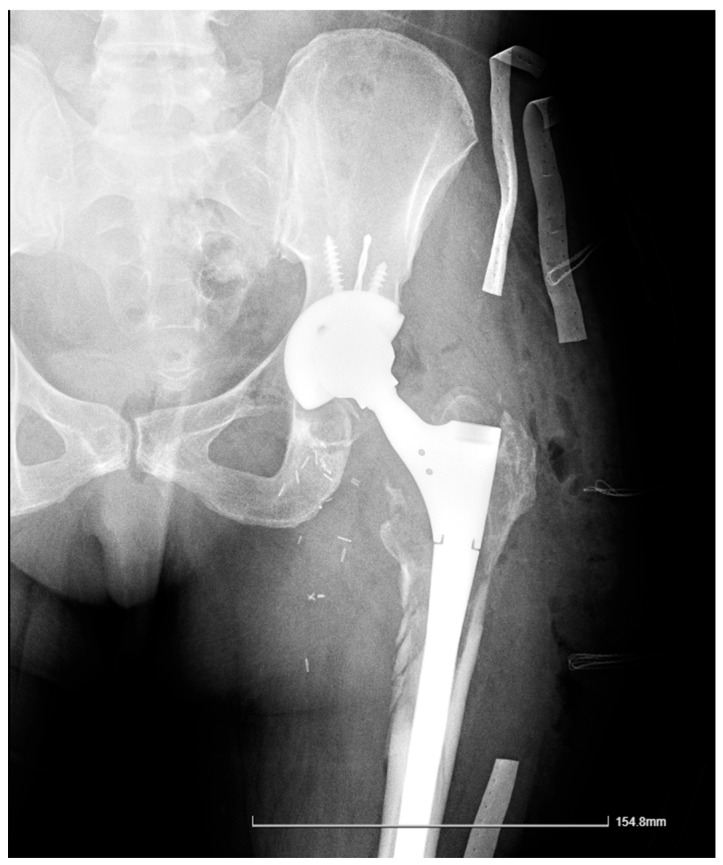
X-Ray of the left pelvis with the modular revision hip prosthesis in situ.

Two weeks after the initial procedure, a reintervention was performed, during which the original prosthesis was removed, visible metallic debris was debrided, and two alpha-defensin tests (Synovasure, Warsaw, USA^®^) were performed, both of which returned negative results 15 min after the sample collection. Intraoperatively, the stem was found to be extensively osteointegrated, and the acetabular was well fixed. The polyethylene liner showed signs of wear and the head–neck junction appeared loose, with minor signs of corrosion, but it was stable. The negative alpha-defensin results further supported the exclusion of PJI, and an exchange arthroplasty was performed with a modular hip stem, ceramic head, polyethylene liner, and a standard cup which was fixed with two posterosuperior screws. A postoperative radiograph is shown in Figure 8.

### 3.6. Outcome

Immediate outcomes: The postoperative radiograph demonstrated a well-fixed prosthesis, and wound healing was uneventful without any systemic complications. The patient initiated rehabilitation and was subsequently discharged.

**Figure 9:**
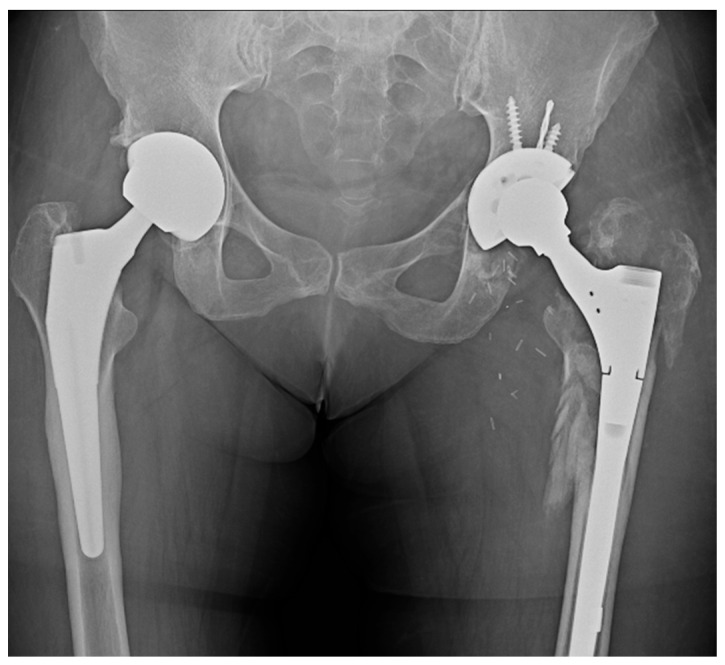
X-Ray of the pelvis at the 6-month mark. A healthy callus is shown forming on the medial side of the femur.

Six months postoperative: The patient presented with no further local complications, with a well-healed scar, normal hip range of motion, and mild hip pain. Notably, slight stem subsidence could be seen, with consequent limb shortening of 1 cm, and this was managed with orthotic footwear and it was accompanied by moderate bilateral knee pain. MRI evaluation of the knee revealed degenerative changes, which were managed conservatively with intra-articular injections of platelet-rich plasma (PRP), hyaluronic acid (HA), and rehabilitation, resulting in resolution of the subjective complaints. Follow-up radiographs at the six-month mark demonstrated a stable prosthesis without evidence of loosening or lytic changes, as shown in Figure 9.

## 4. Discussion

### 4.1. Comparison with Existing Literature

The incidence of asymptomatic ARMD is substantially higher than symptomatic ARMD, with MRI-based evaluations at 43% in MoM and 41% in MoP, with pseudotumors being present in 37% of articulations. In the MoP cohort, the mean pseudotumor volume was 20 mL, with the maximum being 200 mL [23].

Clinical failures of THA due to trunnionosis have increased substantially in the past decade, with an estimated 0.01 to 3% of THA failures being attributed to this etiology. Several implant design features are implicated in the trunnionosis process, including a tapered design, the surface topography, neck and taper flexural rigidity, head size, and head-trunnion material [7]. Unexplained pain in patients with well-positioned MoM hips warrants further investigation with MRIs to identify features predictive of ALVAL [24].

Certain metal-head couplings have been characterized by high failure rates, as highlighted by specific surveillance for implantable medical devices, including the “Rejuvenate” THA system and the Zimmer M/L Taper Style [25,26]. In these cases, surveillance should be activated promptly to initiate a recall procedure and ensure close observation of the implanted device.

A larger implantation duration and larger head size are factors identified as contributing to higher corrosion grades, while older age, stainless steel stems, and indications for revision due to other factors (infection, fracture, or loosening) are associated with lower corrosion grades [27]. Beyond these reasons, manufacturer-specific causes influence the incidence of trunnionis. These include suboptimal metallurgy, inadequate corrosion shielding, and a poor surface finish. Additionally, in some cases, surgeons use metal heads from different manufacturers than the stems, thus leading to potential coupling issues despite the 12/14 taper standard.

Clinical outcomes following rTHA for trunnion corrosion are poor, with 26% of patients undergoing re-revision at a mean length of 8.0 months, most frequently for instability and infection. The survival rate of the prosthesis was 75% at 2 years and 73.2% at 5 years [28].

For MoM bearings, the clinical outcomes are similarly poor. A systematic review reported a 21% re-revison rate with mean intervals of 21 and 61 months. The most common complications and indications for re-revision were dislocation, ARMD recurrence, and acetabular loosening [29]. The risk of serious complications following MoP THA for ARMD is considerable. One study reported 45% of patients developing complications, of which 18% experienced dislocation, 18% presented with persistent pain, and 9% developed PJI [30].

### 4.2. Clinical Implications

In this case, both the ESR and C-reactive protein levels were elevated preoperatively (ESR = 40 mm/1 h and CRP = 30 mg/L). Given the suspicious clinical aspect of the local tissue, the decision to not perform a single-stage revision was warranted. Negative microbiological cultures, a negative alpha-defensin test, and the histological analysis, all collected during the first intervention, demonstrated the absence of neutrophil infiltration, which effectively excluded both PJI and culture-negative PJI, allowing for safe a rTHA to be performed after the histological confirmation of ARMD.

The additional serum values associated with ARMD include cobalt and chromium ion levels. The diagnostic capability of preoperative serum cobalt and chromium levels in distinguishing high-grade ALVAL from low-grade ALVAL is notably high, particularly in total knee arthroplasty, and it has also demonstrated moderate utility in total hip arthroplasty [7]. There is evidence to suggest that a differential elevation of cobalt, more than chromium, is often seen in trunnionosis. A serum cobalt-to-chromium ratio of >1.4 has been reported to have a 93% sensitivity and 70% specificity for trunnion corrosion [31].

Plain radiographs provide limited information when evaluating symptomatic THAs suspected of ALVAL and have been found to reveal no evidence of osteolysis and only some demineralization [8]. A study evaluating the ARMD of large-sized femoral head MoM THAs with the use of CT showed a 39% incidence of pseudotumor formation [32]. The most accurate way of determining soft-tissue abnormalities remains the MRI, with MARS-MRIs proving especially useful in patients with THA. An MRI is able to best determine the volume, location, and consistency of a pseudotumor as well as associated soft-tissue and bony pathology [33].

Flow cytometry analyses have revealed that the peri-implant pseudotumor tissues express two principal phenotypes, namely, macrophage-dominated and T-lymphocyte-dominated responses. In macrophage-dominated inflammation, the average cellular composition is 54% macrophages and 25% T-lymphocytes, whereas in T-lymphocyte-dominated responses, the average is 20% macrophages and 54% T-lymphocytes [34].

Specific genetic factors influence the incidence of ALVAL. Certain HLA genotypes are associated with reduced risk, while others, such as DQB1∗05:03:01 and DRB1∗14:54:01, may have clinical significance for preoperative screening and in guiding prospective surgical decision-making [35].

**Figure 10:**
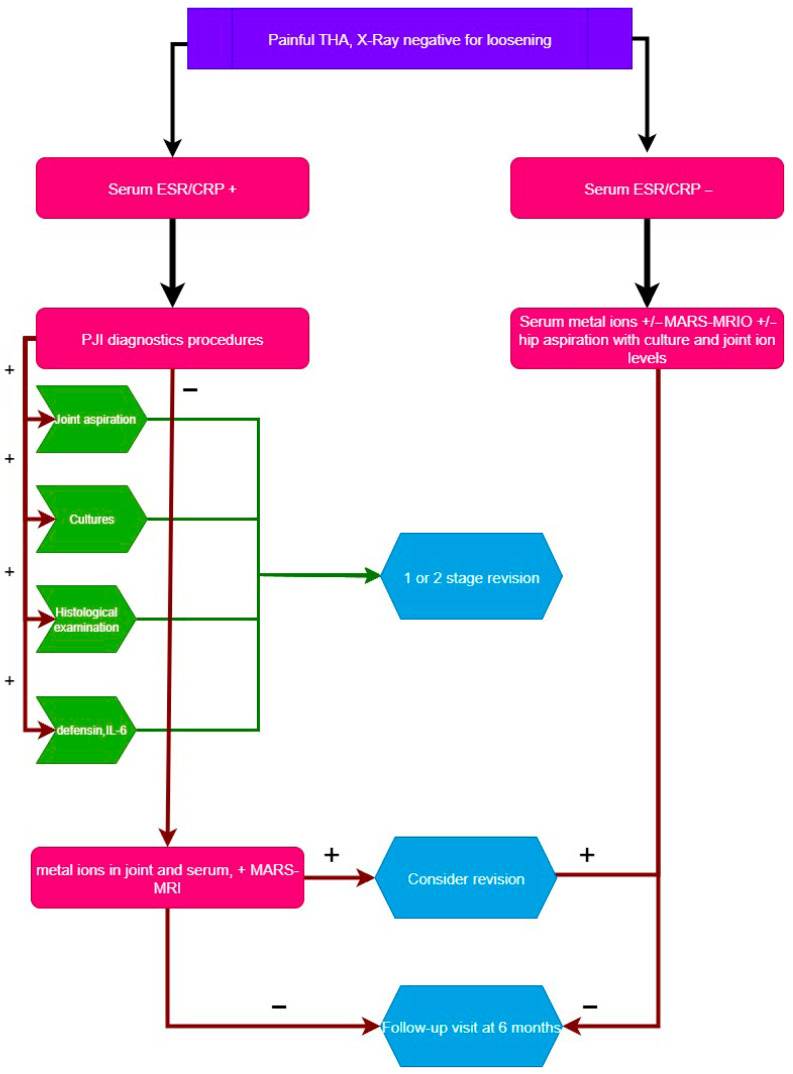
Proposed algorithm illustrating establishing the diagnosis for trunnionosis (ESR, erythrocyte sedimentation rate; CRP, C-reactive protein; MARS-MRI, metal artifact reduction sequence magnetic resonance imaging. Sourced from [3] with proposed changes (labeled with green in the flowchart).

As ARMD represents a diagnosis of exclusion, it is essential to conduct a comprehensive examination of all potential causes of prosthesis failure. The consequence of untreated PJI is catastrophic, particularly following revision arthroplasty, and it frequently leads to the loss of the newly implanted prosthesis and substantial patient mortality and morbidity. We proposed an updated algorithm for the diagnosis of trunnionosis and the exclusion of PJI, as illustrated in Figure 10.

Currently, evidence regarding definitive treatment guidelines for patients with trunnionosis is limited. Nonoperative treatment has been advocated for patients with mild and transient symptoms, with reevaluation at 6 months.

For patients with confirmed diagnoses, after the exclusion of infection, the next step is revision THA [3]. The prosthetic liner should be exchanged to avoid residual metal debris, and the metallic head should be exchanged with a ceramic one [36]. If the taper is severely damaged or the stem is loose, revision of the femoral stem is also indicated.

### 4.3. Limitations

This study had several limitations. The paucity of high-quality evidence on the subject is notable, as most available studies are classified as level III or IV evidence. A lack of standardized definitions further complicates the literature, with terms such as metallosis, trunnionis, ARMD, and ALVAL all being adequate terms in describing the pathology. These factors contribute to inconclusive results and hinder the identification of the most up-to-date evidence-based treatment options. Currently, no clinically validated treatment algorithm exists, and most available studies lack sufficiently large cohorts to adequately assess the validity of risk factors, treatment strategies, clinical outcomes, and long-term results. Consequently, there is a critical gap in the literature regarding standardized diagnostic criteria and evidence-based guidelines, which limits the ability to compare studies and develop consensus-driven management protocols for patients affected by these conditions. As awareness of the condition increases, further studies are required to accurately determine its the true incidence, identify associated risk factors, and establish optimal management strategies encompassing diagnosis, treatment approaches, and long-term outcomes.

## 5. Conclusions

Despite recent advancements in prosthesis design, ARMD can still occur, even in metal-on-polyethylene implants, and it should be considered in the differential diagnosis of chronic hip pain after THA. A thorough diagnostic workup should be undertaken, including multiple cultures, histological analysis, sonication of the removed implant, and emerging paraclinical tests such as an alpha defensin assay.

ARMD remains a rare complication of THA, occurring in approximately 5% of patients with MoM prostheses and approximately 3% of those with MoP prostheses, and it is mainly attributed to trunnionosis. Revision surgery for trunnionosis is increasing, with reported rates ranging from 0.1% to 3%. Clinical outcomes after revision have been poor, with a 75% prosthesis survival rate at five years, which is mainly attributed to infection, fracture, and persistent pain.

## Data Availability

Data availability is subject to hospital approval.

## Supplementary Materials

The following supporting information can be downloaded at: https://www.mdpi.com/article/10.3390/reports8040214/s1, Video S1: Intraoperative drainage of the pseudotumor.

## Abbreviations

The following abbreviations are used in this manuscript:

ARMD	adverse reaction to metal debris
ALVAL	acute lymphocytic vasculitis-associated lesions
MoP	metal on polyethylene
MoM	metal on metal
THA	total hip arthroplasty
rTHA	revision total hip arthroplasty
PJI	periprosthetic joint infection
ESH	erythrocyte sedimentation rate
CRP	C-reactive protein
